# Supplementary material for: Chromothripsis during telomere crisis is independent of NHEJ, and consistent with a replicative origin
Source: Genome Res. 2019 May;29(5):737–49. doi: 10.1101/gr.240705.118 (PMC6499312; doi:10.1101/gr.240705.118)
Supplement: Supplemental Material [file supp_gr.240705.118_Supplemental_file_1.zip › contigs/annotated_contigs/DB73/contig.3.DB73_length_689_mean_cov_7.4702467344.docx]

**DB73_length_689_mean_cov_7.4702467344**

```
CGGCGCCATCTCAGTCGCCACACCCTCCGCCTCTTGGCTTCGAGGATTCTCATGCCTCAGCCTCCTGTGTAGCTGGGACTACAGGTGCA
 >chr16:23656159-23656422 + E=1e-135
CGCCACTACACTGGGCTAATTTTTGTATTTTTAGTAGAGGCAGGGTTTTGCCACGTTGGCCAGGCTGGTCTCAAACTCTTGGCCTCAAG

TGATCCACCTGCCTTGGCCTCTCAAAGTACTGGGATTACAAGTGTGAGCCACTGCACCCAGCCTAACTATATATATTATATATA|GGCG

GGCGGGCGG|ATCACTTGAGGTCAGGAGTTCAAGACCATCCTGGCCAACATGGTGAAACCCGGTCTCTACTAAAAATACAAAAAAAAAA
          >chr16:23661667-23661887 - E=2e-110
AAAATTAGCCAGGTGTGGTGGGAGGAAGAGGTGGTGTGTGGGGAGGGAGGGAGGCAGGAGAATCGCTTGAACCTGGAGGTTGCAGTGAG

TCAAGATCGCACCATTACACTCCAGCCTGGGCAATAAGAGCAAAACT|CC|TCAGTTGATCCACCTTCCTTGGCCTCCCAAAGTGCTGG
                                                >chr16:23635069-23635268 + E=6e-108
GATTACAGGCGTGAGCCACTGCGCCCAGCCTAGGTTCACATTTTAAATAGAAACTTCAGTCAGGGATGTCATTCTATTGATTTTTTTTT

AAACAAATCTCTCTCTCTTTAGATCTTTCAGATTCTTTCAAGACTCAAGCCTAGGTTATTACCTGCACTT
```
